# In Situ Preconditioning of Human Mesenchymal Stem Cells Elicits Comprehensive Cardiac Repair Following Myocardial Infarction

**DOI:** 10.3390/ijms22031449

**Published:** 2021-02-01

**Authors:** Woo-Sup Sim, Bong-Woo Park, Kiwon Ban, Hun-Jun Park

**Affiliations:** 1 Department of Biomedicine & Health Sciences, The Catholic University of Korea, 222 Banpo-daero, Seocho-gu, Seoul 137701, Korea; woosup269@naver.com (W.-S.S.); bwpark@catholic.ac.kr (B.-W.P.); 2 Division of Cardiology, Department of Internal Medicine, Seoul St. Mary’s Hospital, The Catholic University of Korea, 222 Banpo-daero, Seocho-gu, Seoul 137701, Korea; 3 Department of Biomedical Sciences, City University of Hong Kong, Tat Chee Avenue, Kowloon, Hong Kong, China; 4 Cell Death Disease Research Center, College of Medicine, The Catholic University of Korea, Seoul 06591, Korea

**Keywords:** human mesenchymal stem cells, hepatocyte growth factor, engineered mesenchymal stem cells, myocardial infarction, vascular regeneration

## Abstract

Human bone marrow-derived mesenchymal stem cells (BM-MSCs), represented as a population of adult stem cells, have long been considered as one of the most promising sources for cell-based cardiac regenerative therapy. However, their clinical use has been significantly hampered by low survival and poor retention following administration into failing hearts. Here, to improve the therapeutic effectiveness of BM-MSCs, we examined a novel therapeutic platform named in situ preconditioning in a rat myocardial infarction (MI) model. In situ preconditioning was induced by a combinatory treatment of BM-MSCs with genetically engineered hepatocyte growth factor-expressing MSCs (HGF-eMSCs) and heart-derived extracellular matrix (hdECM) hydrogel. Subsequently, our results demonstrated that in situ preconditioning with cell mixture substantially improved the survival/retention of BM-MSCs in the MI-induced rat hearts. Enhanced retention of BM-MSCs ultimately led to a significant cardiac function improvement, which was derived from the protection of myocardium and enhancement of vessel formation in the MI hearts. The results provide compelling evidence that in situ preconditioning devised to improve the therapeutic potential of BM-MSCs can be an effective strategy to achieve cardiac repair of MI hearts.

## 1. Introduction

Ischemic heart disease, such as myocardial infarction (MI), has long been the single largest leading cause of deaths and disabilities worldwide [1]. Due to exceedingly minimal self-regenerative potential in the heart as well as limited therapeutic options, cell-based cardiac regeneration therapy has emerged as one of the most promising alternatives for treating damaged hearts [2]. Among several candidates, due to proven safety, the convenience of acquisition and maintenance, and notable paracrine effects to secrete anti-apoptotic and angiogenic growth factors, the human mesenchymal stem cells (hMSCs) are considered as a more competitive agent as the sources for cell-based cardiac regeneration therapy [3,4]. The results from several preclinical studies using animal models have demonstrated that the administration of hMSCs into the MI hearts exhibited a capability to engraft within a heart, decrease the infarct size, and improve contractile function [5,6]. Subsequently, multiple clinical trials for assessing the possibility of using hMSCs as cellular agents for treating heart disease were conducted worldwide [7]. While some of these trials have demonstrated moderate improvement of left ventricular function, most clinical trials failed to report significant functional enhancement following the administration of hMSCs [8,9,10,11].

The possible reasons why the implantation of hMSCs could not yield successful results in multiple clinical trials was their low survival rate and poor engraftment in the failing heart [12]. Indeed, several previous studies demonstrated that transplanted stem cells did not remain in the targeted site for sufficient duration [13]. Thus, during the last two decades, multi-faceted approaches have been developed to improve the therapeutic potency of hMSCs for the treatment of heart disease [14,15]. One of the most well-delineated strategies to promote the therapeutic potential of hMSCs is preconditioning the hMSCs. It has been intensively explored that hMSC preconditioning is achieved by exposing the hMSCs to several applications such as hypoxia, treatment with pharmacological/chemical agents [16], cytokines, or growth factors [17], or physical stimuli including biomaterials [18,19,20,21] under in vitro condition before implantation into the hearts, and there is growing evidence that the preconditioning of hMSCs indeed bolstered therapeutic potentials of hMSCs.

Therefore, in the present study, we sought to develop an efficient strategy coined as in situ preconditioning, which is devised to promote the therapeutic effectiveness of hMSCs through enhancing survival, retention, and engraftment of hMSCs post-transplantation into the MI-induced rat heart. Unlike classic preconditioning, which generally takes place in vitro condition, to simulate and provide preconditioning-like effects more persistently in situ in the hearts, we administered hMSCs derived from human bone marrow mesenchymal stem cells (BM-MSCs) together with genetically engineered hMSCs designed to secrete a hepatocyte growth factor-expressing mesenchymal stem cells (HGF-eMSCs) continuously. To further improve the retention of those intramyocardially injected cells and provide a cardiac tissue-like microenvironment, we encapsulated the cell with the heart-derived extracellular matrix (hdECM) hydrogel. We hypothesized that BM-MSCs could be preconditioned constantly and strengthened by HGF secreted from co-administered HGF-eMSCs within the heart, and those empowered BM-MSCs would demonstrate an enhanced therapeutic potential for cardiac repair. Subsequently, we observed that our in situ precondition strategy exhibited a greater survival and retention of BM-MSCs within the MI-induced hearts, which led to a significant improvement in cardiac function and an enhancement of vessel formation after MI. The results obtained from the in situ preconditioning strategy designed to improve the therapeutic potential of BM-MSCs have important implications for future stem cell therapies in heart repair.

## 2. Results

### 2.1. Cellular Characteristics of HGF-eMSCs

**Figure 1:**
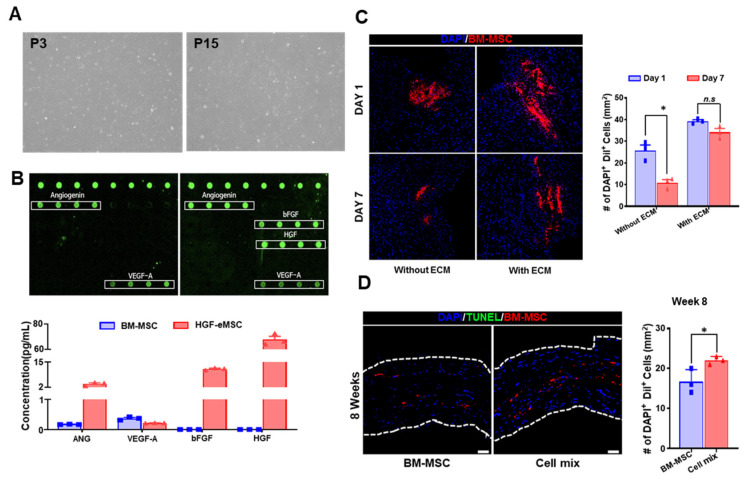
In situ preconditioning promoted the retention of bone marrow-derived mesenchymal stem cells (BM-MSCs) administered into the ischemic myocardium. (**A**) Morphology of hepatocyte growth factor–expressing MSCs (HGF-eMSCs). (**B**) Paracrine factor secretion properties of HGF-eMSCs. The cytokine array kit incubated with conditioned media of HGF-eMSC cultures collected on day 7 verify the presence of several paracrine factors. (**C**) Encapsulation of BM-MSCs with 0.5% porcine heart-derived extracellular matrix (hdECM) improved the retention of BM-MSC in the myocardial infarction (MI)-induced hearts. Representative images of BM-MSC stained with red-fluorescence dye and their quantification summary. * *p* < 0.05 compared to day 1 group. (**D**) In situ preconditioning, which co-administrated the BM-MSCs and HGF-eMSCs with hdECM, significantly augmented the retention of BM-MSCs. The representative images of 1,1′-Dioctadecyl-3,3,3′,3′-Tetramethylindocarbocyanine Perchlorate (DiI)-positive BM-MSC in the 8 weeks infarct zone and their quantification summary in three areas. Scale bar: 50 µm. Cell mix: BM-MSCs + HGF-eMSCs + hdECM. * *p* < 0.05 compared to BM-MSC group.

We previously demonstrated that the HGF-eMSCs were identical to typical BM-MSCs [15]. HGF-eMSCs exhibited major cellular characteristics of hMSCs, including homogeneous spindle-like cell morphology (Figure 1A) and significant expressions of several specific markers for hMSCs such as CD44, CD90, and CD105. The HGF-eMSCs also exhibited high proliferative potential and released paracrine factors, including angiopoietin 1 (Ang) and vascular endothelial growth factor (VEGF). Most importantly, HGF-eMSCs secreted a substantial concentration of human basic fibroblast growth factor (bFGF) and HGF proteins compared with BM-MSCs determined by a cytokine array kit (Figure 1B).

### 2.2. Combinational Cell Therapy Improves Cell Retention in Ischemic Myocardium

To examine whether hdECM could augment the survival/retention of hMSCs in the MI-induced hearts, we intramyocardial injected the hMSCs into the myocardium of BALB/c nude mouse, in which MI was induced by ligating the left anterior descending (LAD) coronary artery in the absence or presence of hdECM (0.5%). To trace the injected hMSCs within the heart tissues, hMSCs were pre-labeled with a potent red fluorescence dye, CM-DiI, before cell injection (Figure 1C). Interestingly, microscopic observations of mouse heart tissues harvested 7 days post-implantation showed substantially higher numbers of hMSCs in hearts that received hMSCs with hdECM than those treated with hMSC only. These results indicated that hdECM significantly promoted the retention of implanted hMSCs, and thus, we decided to continuously use the hdECM to implant the hMSCs into the hearts in further experiments (Figure 1C).

Next, we explored our central hypothesis of whether dual implantation of BM-MSC with HGF-eMSCs (cell mixture hereafter) can bolster the survival and retention of BM-MSC in ischemic hearts. Hearts were harvested 8 weeks after implanting the cells either BM-MSC alone (5 × 10^5^) or a cell mixture (2.5 × 10^5^ of BM-MSCs and 2.5 × 10^5^ of HGF-eMSCs) to compare the cell retention rates between two groups. As a result, while a smaller number of BM-MSC were injected in the cell mixture group compared with the BM-MSC only group, the cell mixture-treated hearts retained a substantially greater number of cells in the myocardium than other hearts. Moreover, terminal deoxynucleotidyl transferase dUTP nick end labeling (TUNEL) analysis results further demonstrated that most of the cells remaining in the MI hearts were viable, verifying that mixed treatment with HGF-eMSCs considerably enhanced the survival and retention of BM-MSCs in failing hearts (Figure 1D).

### 2.3. Combinational Cell Therapy Improves Cardiac Function after Myocardial Infarction

Finally, to investigate whether the simultaneous administration of BM-MSCs with HGF-eMSCs can amplify the therapeutic effects BM-MSCs, we established the following experimental groups: (1) MI control group receiving hdECM (0.5%) only, (2) BM-MSCs (5 × 10^5^) only, (3) BM-MSCs (5 × 10^5^) only, and (4) Cell mixture: BM-MSCs (2.5 × 10^5^) and HGF-eMSCs (2.5 × 10^5^). The cells were well suspended in the 50 μL of Phosphate-buffered saline (PBS) buffer containing 0.5% hdECM.

**Figure 2:**
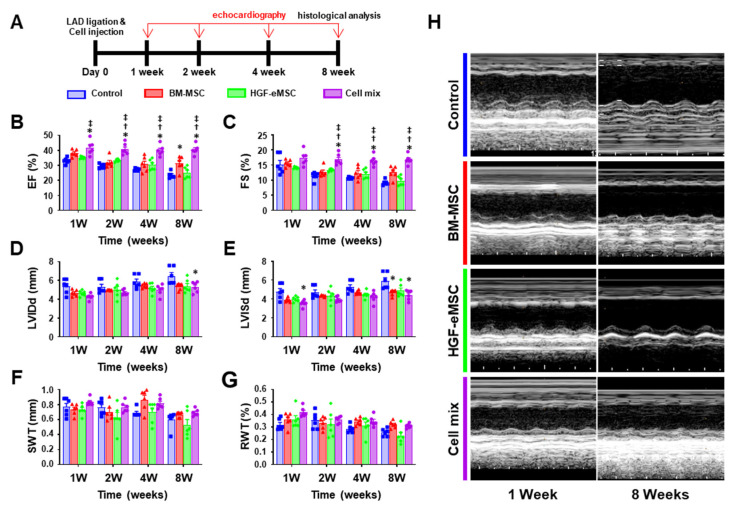
In situ preconditioning improved cardiac function following myocardial infarction. (**A**) Schematic diagram of an in vivo experimental schedule including the time point of echocardiography in a mice model. (**B**–**G**) Summary of left ventricular eject fraction (EF), fractional shortening (FS), left ventricular internal diastolic dimension (LVIDd), left ventricular internal systolic dimension (LVISd), septal wall thickness (SWT), and relative wall thickness (RWT) at 8 weeks post-intervention. (**H**) Representative echo images of all experimental groups at 8 weeks after left anterior descending (LAD) ligation. * *p* < 0.05 compared to control group, ^†^ *p* < 0.05 compared to BM-MSC group; ^‡^ *p* < 0.05 compared to HGF-MSC group. *n* = 6 per each group.

Then, we intramyocardially injected the individual group of cells into the MI-induced hearts. To exclude any potential risk of genetically modified HGF-eMSCs, we irradiated the HGF-eMSCs with 100-Gy radiation before administration into the hearts. We previously observed that irradiated HGF-eMSCs markedly reduced their proliferation potential both in vitro and in vivo conditions. We also previously verified that HGF-eMSCs did not give rise to tumor formation in the heart tissues. Subsequently, to monitor the recovery of left ventricular function and evaluate the cardiac remodeling, we executed serial echocardiography on a regular basis until 8 weeks post-interventions (Figure 2A). We decided to monitor the hearts until 8 weeks post-interventions because the heart continues to undergo adverse cardiac remodeling for a relatively long time after myocardial infarction. Thus, we sought to follow up the cardiac function until 8 weeks after interventions.

Ejection fraction (EF) and fractional shortening (FS), the primary indicators of cardiac contraction, were substantially greater in the cell mix-treated hearts until week 8 compared to other experimental groups. Although the BM-MSCs only administered hearts exhibited a trend of cardiac function improvement at week 8, it was still lower than the cell mix administered group (Figure 2B,C). Moreover, several parameters for cardiac remodeling such as left ventricular internal diastolic dimension (LVIDd), left ventricular internal systolic dimension (LVISd), septal wall thickness (SWT), and relative wall thickness (RWT), indicate that the degree of adverse cardiac remodeling in the hearts receiving cell mix was substantially lower compared to other experimental groups (Figure 2D–H).

### 2.4. Combinational Cell Therapy Improves Host Angiogenesis through Long-Term Paracrine Effects

**Figure 3:**
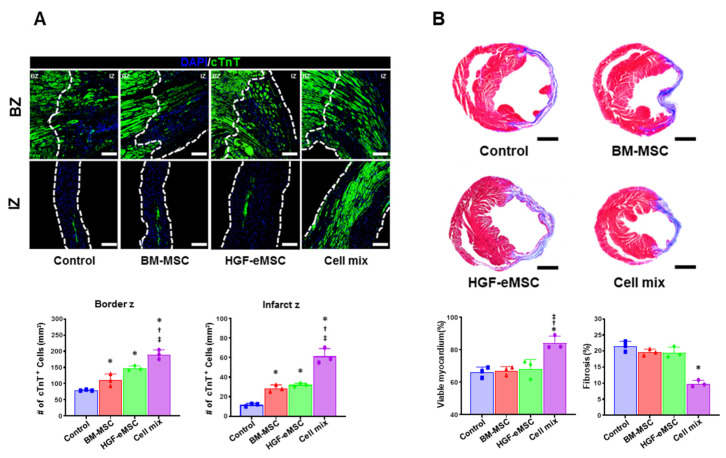
In situ preconditioning protected the myocardium and reduced cardiac fibrosis. (**A**) Representative images of cardiac troponin T (cTnT: Green) positive area in the infarct zone and border zone in all experimental groups 8 weeks after cell transplantation (IZ: infarction zone, BZ: border zone, scale bar: 100 µm) and their quantification summary. (**B**) Representative images of Masson’s trichrome staining with the heart tissues harvested 8 weeks after MI and quantification results of viable myocardium (%) and cardiac fibrosis (% of Left ventricular wall) Scale bar: 5 mm. IZ: infarction zone, BZ: border zone. * *p* < 0.05 compared to control group, ^†^ *p* < 0.05 compared to BM-MSC group; ^‡^ *p* < 0.05 compared to HGF-MSC group. *n* = 3 per each group.

Given the higher cardiac function and lower adverse cardiac remodeling in the hearts receiving cell mixture, to investigate the possibility of whether the dual cell treatment can directly protect the myocardium from ischemic insult, we quantified the viable myocardium in the cardiac tissues harvested from all experimental groups at 8 weeks post-MI by using the cardiac troponin T (TNNT2) antibody. As a result, we observed a significantly higher number of TNNT2-positive cardiomyocytes in the cell mixture group than other experimental groups (Figure 3A). Furthermore, the results from Masson’s trichrome staining using heart tissue collected at 8 weeks post-MI showed that cell mixture-treated hearts exhibited a significantly lower level of cardiac fibrosis than other experimental groups (Figure 3B).

**Figure 4:**
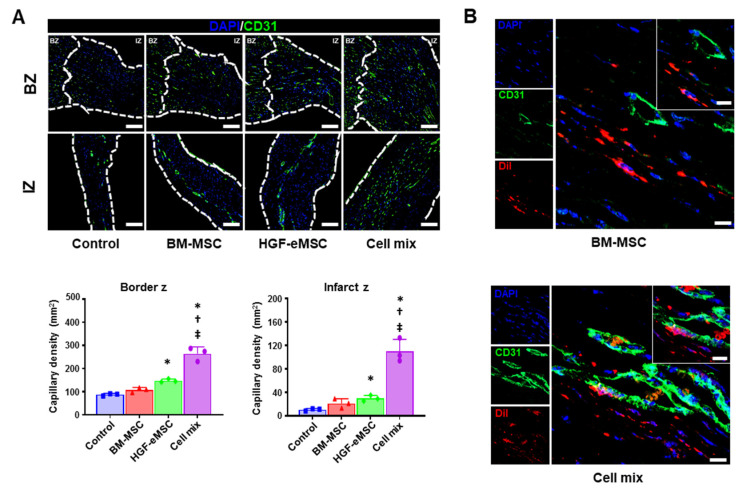
In situ preconditioning improved vascular regeneration in the ischemic hearts. (**A**) Representative images of CD31-positive capillaries in the infarct zone and border zone 8 weeks after cell transplantation (IZ: infarction zone, BZ: border zone, scale bar: 100 µm) and their quantification summary of the capillary density in all experimental groups. * *p* < 0.05 compared to control group, ^†^ *p* < 0.05 compared to BM-MSC group; ^‡^ *p* < 0.05 compared to HGF-MSC group. *n* = 3 per each group. (**B**) Representative images of 1,1′-Dioctadecyl-3,3,3′,3′-Tetramethylindocarbocyanine Perchlorate (DiI)-positive BM-MSCs in the heart tissues receiving BM-MSCs alone or cell mixture at 8 weeks post-treatment. Scale bar: 10 µm. IZ: infarct zone, BZ: border zone.

It has been clearly recognized that angiogenic paracrine factors released from BM-MSCs are critical effectors to induce vascular regeneration in the ischemic tissues. Thus, to confirm the angiogenic potential of combinational cell therapy, we performed immunostaining with CD31 antibody, which is a well-known marker specific for endothelial cells, using the cardiac tissues harvested from all experimental groups at 8 weeks post-MI. Histological images showed that the number of capillaries (mm^2^) in the border zone and infarct zone of the hearts from the cell mix group was significantly higher than in other groups (Figure 4A). Of note, the cell mix group directly contributed to the vasculogenesis, possibly through the differentiation of BM-MSCs into the endothelial cell (Figure 4B). Collectively, these results clearly indicate that the cell mixture treatment led to comprehensive cardiac repair through the improvement of cardiac function, reduction of cardiac fibrosis, and enhanced vascular regeneration.

## 3. Discussion

Disappointingly, multiple clinical trials recently conducted to test the therapeutic efficacy of hMSCs against human heart failure patients worldwide have not been able to repeat the positive outcomes of animal models that have been observed in many successful preclinical studies. Consequently, a newly formulated direction particularly in the adult stem cell research area has been focusing on developing more effective strategies to promote the therapeutic potential of hMSCs. As a part of it, in the present study, we intended to examine a combinatory cell therapy using BM-MSCs and HGF-eMSCs, which we named in situ preconditioning strategy, to promote the therapeutic efficacy of hMSCs for the treatment of ischemic heart disease. We demonstrated that the in situ preconditioning platform, a combinational platform consisting of BM-MSCs, HGF-eMSCs, and hdECM hydrogel, successfully preserved cardiac function by enhancing vessel formation and minimizing adverse remodeling in post-MI hearts. Of note, we observed that the majority of intramyocardially injected BM-MSCs stably survived in the infarcted area and were incorporated into neighboring host vasculature, suggesting the enhanced vasculogenic potential of BM-MSCs via HGF-eMSCs.

Despite continued intensive efforts, still, one of the most critical hurdles in cell-based cardiac regeneration therapy is low survival and poor engraftment following transplantation in the failing hearts. It has been clearly shown that a large proportion of the injected cells are lost from the myocardium within the first few minutes after injection, and only a minimal number of cells are retained after weeks [22]. Therefore, enhancing the survival/retention of stem cells is the key to success and remained an essential goal for stem cell-based cardiac regeneration therapy. To promote the survival/retention of BM-MSCs in the ischemic hearts, as a partner to induce in situ preconditioning, we chose HGF-eMSCs consistently releasing HGF. HGF is a well-known multifunctional factor that has been known to stimulate angiogenesis, inhibit fibrosis, and reduce apoptosis [23,24,25,26]. A recent study from Wu et al. also evidenced that a combinatory implantation of MSCs with HGF-modified MSCs and small-molecular hydrogel significantly improved cardiac function and reduced scar size [27].

To bolster the survival and retention of BM-MSCs, we additionally employed the hdECM hydrogel derived from porcine heart tissue. Among many distinct types of biomaterials, we selected hdECM because of its several unique advantages. As natural biomaterial obtained from heart tissues, hdECM consists of proteins and polysaccharides specific for heart tissue, and these properties allowed recreating a more complex biochemically relevant microenvironment, mimicking heart-specific extracellular matrix (ECM) composition. It can also more suitably establish the structure and mechanical properties of the tissue as a scaffold. Thus, as a cell carrier, hdECM hydrogel helped provide a favorable microenvironment to a cell mixture of BM-MSCs and HGF-eMSCs, which allowed them to be more resistant against an extremely hostile microenvironment in the infarcted hearts and eventually lead to an improved survival/retention. Well-survived and appropriately colonized BM-MSCs secreted several paracrine factors related to angiogenesis and anti-apoptosis more reliably and effectively into the diseased heart. These beneficial paracrine factors eventually protected the myocardium from ischemic injury and promoted vascular regeneration, leading to a subsequent restoration of cardiac function. These results suggest that hdECM hydrogel is a safe and functional biomaterial to provide a cardiac niche for cell survival and facilitate vascularization and tissue formation in vivo.

**Figure 5:**
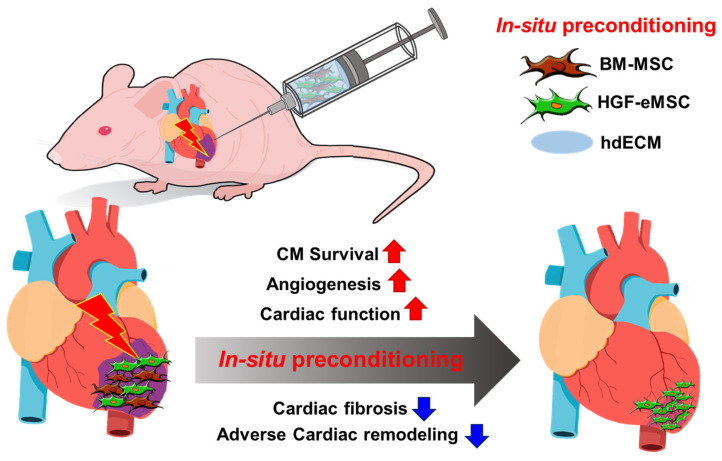
Schematic diagram of the underlying mechanism of in situ preconditioning of BM-MSCs with HGF-eMSCs, and hdECM. BM-MSCs: human bone marrow-derived mesenchymal stem cells, HGF-eMSCs: genetically engineered hepatocyte growth factor-expressing MSCs, and hdECM: heart-derived extracellular matrix. CM: Cardiomyocytes.

In summary, we report a proof-of-concept platform designed to empower the therapeutic potential of BM-MSCs in situ in the heart to effectively treat heart disease. A combinational treatment of BM-MSCs, HGF-eMSCs, and hdECM as a platform to induce in situ preconditioning substantially increased the survival/retention of BM-MSCs through continuous released HGF cytokine (Figure 5). Our results highlight that the effective use of BM-MSCs still has remarkable potential as a source of cell-based cardiac therapy.

## 4. Materials and Methods

### 4.1. Derivation of hMSCs from Human Bone Marrow

BM-MSCs were obtained from the Catholic Institute of Cell Therapy (CIC), Seoul, Korea [28,29]. Human bone marrow aspirates were obtained from the iliac crest of healthy donors aged 20 to 55 years with approval from the Institutional Review Board of Seoul St. Mary’s Hospital (approval numbers KIRB-00344-009 and KIRB-00362-006). The bone marrow aspirate from each donor who consented was collected and sent to the Good Manufacturing Practice (GMP)-compliant facility of the CIC (Seoul, Korea; www.cic.re.kr) for the isolation, expansion, and quality control of human BM-MSCs.

### 4.2. Generation of Engineered HGF-eMSCs

hMSCs engineered to continuously secrete hepatocyte growth factor (HGF-eMSCs) were generated as we previously described [28]. Briefly, we first produced replication-incompetent lentiviral vectors, each containing c-Myc, and hTERT. A gene construct expressing the tetracycline transactivator protein was inserted together to use the Tet-off system. These lentiviral vectors were extracted and quantified from DH5α Escherichia coli cells using the EndoFree Plasmid Maxi Kit (QIAGEN, Hilden, Germany). Lenti-X cell (Clontech Laboratories California, Mountain View, CA, USA) was transduced using Lipofectamine PLUS reagent (Thermo Fisher Scientific, Waltham, MA, USA) to amplify the lentiviruses. Then, the lentiviruses were transduced to BM-MSCs at 100 multiplicity of infection using RetroNectin (Clontech Laboratories). Lastly, Zeocin (500 μg/mL) and puromycin (1 μg/mL) were used to select the cells infected with lentiviruses following infection.

### 4.3. Cytokine Array.

The conditioned medium was harvested from cultured BM-MSC or HGF-eMSC and analyzed using the Human Angiogenesis Array Q1 kit (Raybiotech, QAH-ANG-1-1, Atlanta, GA, USA). Data were acquired on a Microarray scanner (Molecular Devices, GenePix^®^ 4300).

### 4.4. Mouse MI Model and Transplantation of Cells

All animal studies were approved by the Institutional Animal Care and Use Committee (IACUC) of The Catholic University of Korea (Approval number: CUMC-2015-0048-04). Myocardial infarction in the rodent model was induced as we previously described [28,29,30]. Male BALB/c nude mouse (8-week-old, 30–40 g; Orient bio, Seongnam, Korea) anesthetized via inhalation of 2% isoflurane were intubated through the trachea with an 18 G intravenous catheter. Then, the mice were mechanically ventilated with medical-grade oxygen. After the left thoracotomy was performed, MI was induced by the left anterior descending artery’s permanent ligation. Immediately after MI induction, BM-MSC cells, HGF-eMSC cells, or a mixture (BM-MSC and HGF-eMSC) of cells were injected directly into the ischemic area. To trace the BM-MSCs within the heart tissues further, the BM-MSCs were pre-labeled with a red fluorescence dye, CM-DiI, prior to cell injection. Briefly, BM-MSCs were stained using CellTracker™ CM-DiI Dye (Invitrogen™, Carlsbad, CA, USA). BM-MSCs were incubated in the CM-DiI Dye solution (5 µg/mL) for 5 min at 37 °C, followed by additional 15 min at 4 °C. After labeling, BM-MSCs were washed with phosphate-buffered saline (PBS) and re-suspend in serum free medium for further uses.

### 4.5. Echocardiography

Echocardiography was performed to confirm the improvement of cardiac function after treatment. After anesthetizing the mouse with 2% isoflurane, echocardiographic images were obtained using an HP SONOS 7500 ultrasound system equipped with L12-5 linear broadband and a 52 Hz L15-7io linear transducer (Affniti 50 G, Philips). Echocardiography was performed at 1, 2, 4, and 8 weeks after treatment, and the following measurements such as ejection fraction (EF), fractional shortening (FS), left ventricular internal diastolic dimension (LVIDd), left ventricular internal systolic dimension (LVISd), septal wall thickness (SWT), and relative wall thickness (RWT) were taken. Echocardiography was performed in a blinded manner to eliminate bias.

### 4.6. Immunohistochemistry

Immunofluorescence was performed on 4-um-thick paraffin sections. After deparaffinization and rehydration, antigen retrieval with target retrieval solution (Dako, Carpinteria, CA, USA) was performed. The sections were blocked and incubated with diluted primary antibody (Dako) at 4 °C overnight. Mouse anti-TNNT2 (Abcam; 1:200, Cambridge, UK) antibody was used to stain myocardium. Next, the samples were incubated with anti-mouse immunoglobulin G Alexa Fluor 488 (Invitrogen; 1:500) secondary antibody for 60 min at room temperature and then stained with 4′,6-diamidino-2-phenylindole solution (VECTOR, Torrance, CA, USA) for nuclear staining. Images of the heart sections were visualized using an LSM 800 laser scanning microscope with Airyscan processing (Zeiss, Oberkochen, Germany).

### 4.7. Determination of Fibrosis

Masson’s Trichrome staining (Sigma, St. Louis, MO, USA) was performed to determine the fibrotic region of MI hearts. Briefly, three frozen sections were fixed in Bouin’s solution at 56 °C for 15 min in each group. Then, these sections were stained using Weigert’s iron hematoxylin solution for 5 min at room temperature. Then, they were stained with Biebrich scarlet-acid fuchsin solution for 2 min at room temperature. Finally, the sections were counterstained with Aniline Blue for 5 min, followed by incubation in 1% acetic acid for 2 min at room temperature. The collagen fibers appeared blue, and the viable myocardium appeared red. The percent of the area of fibrosis to the area of the entire left ventricular wall was quantified using ImageJ with basic add-ons.

### 4.8. Capillary Density Measurement

The heart was sectioned (4 μm), starting from the top to apex, using a microtome (Leica, RM2255, Hessn, Germany). The number of capillaries was counted by CD31 staining and in five random microscopic fields using a fluorescence microscope (Nikon, Tokyo, Japan) and was expressed as the number of capillaries per square mm tissue area.

### 4.9. TUNEL Assay

TUNEL assays (Thermo Fisher Scientific, Waltham, MA, USA) were performed to evaluate apoptosis. Briefly, five paraffin heart sections at 4-μm-thickness were deparaffinized and rehydrated. Following permeabilization with 0.1% sodium citrate containing 0.5% Triton X-100 for 15 min, the reaction was carried out at 37 °C for 1 h in a dark condition. Images of the heart sections were obtained using an LSM 800 laser scanning microscope with Airyscan processing (Zeiss, Oberkochen, Germany).

### 4.10. Data Analysis

All quantitative data are shown as means ± S.E.M unless otherwise indicated. Statistical differences between two groups were analyzed using a two-tailed Student’s *t*-test. The statistically significant differences among 3 or more groups were also analyzed by analysis of variance (ANOVA) with Bonferroni’s post-hoc analysis by using grapad prism 8 software, San Diego, CA, USA). Results were considered significant when the *p*-value was less than 0.05.

## Data Availability

Not applicable.
